# Prediction of mortality and functional decline by changes in eGFR in the very elderly: the Leiden 85-plus study

**DOI:** 10.1186/1471-2318-13-61

**Published:** 2013-06-18

**Authors:** Gijs Van Pottelbergh, Wendy PJ Den Elzen, Jan Degryse, Jacobijn Gussekloo

**Affiliations:** 1 Department of public health and primary care, Katholieke Universiteit Leuven, Leuven, Belgium; 2 Institute of Health and Society, Université Catholique de Louvain, Brussels, Belgium; 3 Research Foundation Flanders, Brussels, Belgium; 4 Department of Public Health and Primary Care, Leiden University Medical Center, Leiden, the Netherlands

**Keywords:** Very elderly, Population-based study, eGFR, Decrease in eGFR, Mortality, Functional decline

## Abstract

**Background:**

The prevalence of chronic kidney disease is high in the elderly, but the effects of a decrease in the eGFR on mortality and functioning are still unclear. The aim of this study was to determine whether the combination of the eGFR and the eGFR slope is a predictor of mortality and functional decline.

**Methods:**

The eGFR (MDRD equation) and the eGFR slope were calculated. The slope was calculated using four annual eGFR measurements taken from 85 to 88 years of age. Mortality and changes in the Mini-Mental State Examination (MMSE), the Geriatric Depression Scale (GDS-15) and the Activities of Daily Living (ADL) scores were analysed as outcomes from 88 years onwards.

**Results:**

378 persons aged 88 years participating to the Leiden 85 plus study, an observational prospective cohort study in the general population, were included. A combined analysis of the baseline eGFR and the eGFR slope showed that an eGFR of >60 ml/min combined with an eGFR decrease of ≥ 3 ml/min/year and an eGFR of <60 ml/min combined with an of eGFR decrease ≥5 ml/min/year were independent predictors of increased mortality. The baseline eGFR, the eGFR slope and a combination of both factors did not predict changes in the MMSE, GDS or ADL scores between 88 and 90 years.

**Conclusion:**

The combination of the eGFR and the eGFR decrease allows the identification of subgroups of very elderly with increased mortality risks and of subgroups of very elderly with an eGFR of <60 ml/min without an increased risk of mortality.

## Background

Demographic predictions for Western countries estimate that the number of persons aged 80 and over will increase significantly in the next several decades [1]. Knowing which factors correlate with higher mortality within this group would allow stratification into high and low risk groups and could result in different therapeutic approaches for different risk groups.

The prevalence of chronic kidney disease (CKD) in older persons is high and increases with advancing age [2]. The prevalence of CKD (an estimated glomerular filtration rate (eGFR) <60 ml/min/1,73 m^2^) is >38% in community-dwelling older persons aged 70 and over.[3-6] As a result, physicians must treat large numbers of older persons with CKD.

A low eGFR has been associated with higher mortality risk [7,8]. The results of the Cardiovascular Health Study [9,10], a study in persons (mean age 72 years) with relatively high cardiovascular risk, indicate that a decrease in the eGFR is an important risk predictor of mortality. Other studies have reported a relationship between the presence of CKD and a decline in activities of daily living (ADL) in addition to this increase in mortality [11,12].

However, whether the eGFR slope or the combination of the eGFR and the eGFR slope is a good predictor of mortality or of a decline in functional performance in the very elderly in the general population remains unknown. Being able to distinguish high risk from low risk patients is a necessary step before testing the effect of interventions in elderly with CKD. In this study, we investigated whether the combination of the eGFR and the eGFR slope predicted mortality and functional decline in the very elderly in the general population at large.

## Methods

### Study population

The present study was carried out as part of the Leiden 85-plus Study, a population-based prospective follow-up study of 85-year-old inhabitants of Leiden, the Netherlands. The study population has been described in detail previously [13]**.** Between September 1997 and September 1999, all inhabitants of Leiden in the 1912–1914 birth cohort were eligible for study participation. No individual was excluded on the basis of health, cognitive function or living situation. All participants gave their informed consent to take part in the study. For those who had severe cognitive impairment, informed consent was obtained from a proxy. The Medical Ethics Committee of the Leiden University Medical Center approved the study.

Participants were visited annually (from age 85 through 90 years) at their place of residence to gather information in face-to-face interviews and to obtain venous blood samples.

In the present study, the baseline eGFR was obtained at the age of 88 years, and the eGFR slope was calculated using the four creatinine measurements taken from age 85 to age 88.

### Primary study parameters

#### *eGFR and eGFR slope*

At study entry and at yearly intervals thereafter, the serum creatinine and serum albumin concentration was measured automatically according to the Jaffe method (Hitachi 747; Hitachi, Tokyo, Japan). The eGFR was calculated using the 4-variable MDRD equation [14]*.* All eGFR values in are expressed in ml/min/1.73 m^2^ but are noted as ml/min. A simplified version of the CKD classification system of the US National Kidney Foundation [15] was used to divide the study population into three eGFR-based groups (≥60, between 45 and 59 and < 45 ml/min/1.73 m^2^).

The slope of the eGFR (ml/min/1.73 m^2^/year) over a period of 3 years for every participant was calculated by the ordinal least square method using exactly 4 eGFR values (at ages 85, 86, 87 and 88 years). Patients were divided in subgroups based on the slope of the change in the eGFR between age 85 and age 88 using predefined subgroups of >-1, -3 to -1, -5 to -3 and < -5 ml/min/1.73 m^2^/year. All eGFR slopes in this article are expressed in ml/min/1.73 m^2^/year but are noted as ml/min/year.

#### *Mortality*

Mortality data, recorded from the start of the study until Feb. 1, 2010, were obtained from the municipal registry. Causes of death were obtained from Statistics Netherlands and are coded according to the International Classification of Diseases and Related Disorders, 10th revision. Causes of death were divided into cardiovascular causes (codes I00–I99) and non-cardiovascular causes (all other codes).

#### *Functional status*

Cognitive function was measured using the Mini-Mental State Examination (MMSE) [16]. The MMSE scores range from 0 to 30, with lower scores indicating impaired cognitive function. Depressive symptoms were assessed using the 15-item Geriatric Depression Scale (GDS), [17]. Scores range from 0 (optimal) to 15. The GDS was only administered to participants who had a Mini-Mental State Examination score greater than 18 points.

Disability in daily living was measured with the Activities of Daily Living (ADL) Scale, which assesses an individual’s competence by combining the scores for 9 activities of daily living. [18]. Higher ADL scores indicate more disability.

#### *Comorbidity*

We obtained information on the presence of disease at baseline from the participants’ general practitioners, nursing home physicians, pharmacy records, laboratory findings and ECGs. The diseases of interest included stroke, myocardial infarction (MI) and diabetes mellitus. We considered diabetes mellitus to be present if diagnosed by the treating physician, if the non-fasting glucose level was greater than 11.0 mmol/L, or if a participant was taking anti-diabetic medication.

#### *Other parameters*

During the interview, we obtained information on other variables, including sex, weight, level of education, income and residence in a nursing home.

### 3 Statistical analyses

Baseline differences between the three eGFR-based groups at age 88 were assessed using the Chi square test for categorical parameters, one-way ANOVA for normally distributed variables or the Kruskal-Wallis test for non-normally distributed variables.

A Cox proportional hazards model was used to study the relationship between mortality from age 88 onwards and the different eGFR and eGFR slope categories. Because we focused on the predictive value of the eGFR and the eGFR slope and not on the underlying aetiology, we present Cox proportional hazards models only including gender. When the eGFR slope was used to divide participants into subgroups, we adjusted for gender and the mean of the eGFRs at ages 85 and 88. The mean of the eGFRs was used to correct for differences in the eGFR at baseline and regression to the mean effects. For all subgroups, the HRs for total, cardiovascular and non-cardiovascular mortality were calculated relative to the participants with an eGFR slope of >-1 ml/min/year. Two sensitivity analyses were performed correcting for gender, history of MI and eGFR at baseline (sensitivity analysis 1) and gender, history of MI and change in weight between the age of 85 and 88 (sensitivity analysis 2).

The relationships between the eGFR, the eGFR slope and the combination of both factors and the mean changes in the MMSE, GDS or ADL scores between age 88 and 90 were evaluated. Differences in the changes in these scores between different subgroups based on the eGFR and the eGFR slope were analysed with one-way ANOVA.

All analyses were performed with SPSS software (version 19.0 SPSS Inc., Chicago, USA).

## Results

### Study population

**Figure 1:**
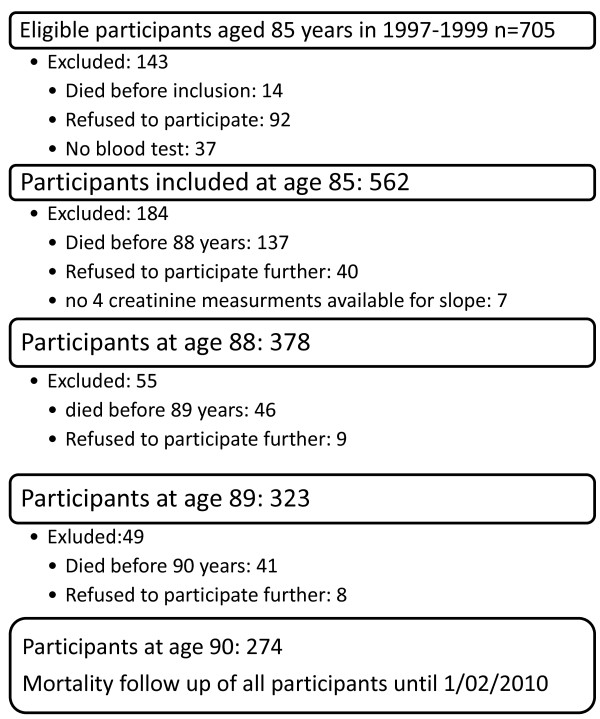
Flow chart of the Leiden 85-plus study.

Between September 1997 and September 1999, 705 inhabitants of Leiden reached the age of 85 years and were eligible for participation in the study. Fourteen individuals died before enrolment in the study, and 92 refused to participate. Of those enrolled in the study, 7 died before blood samples could be collected, and 30 were unwilling to provide a blood sample. As a result, blood samples from 562 subjects were available for analysis at age 85. Figure 1 shows the number of participants at the outset of the study and annually thereafter over the 5-year follow-up period. Blood samples from 378 participants at 85, 86, 87 and 88 years were available for the calculation of the slope of the change in the eGFR between 85 and 88; these participants were included in the present study. The characteristics of the 184 participants who died before age 88 (n=137) refused to participate before the age of 88 (n=40) or had no four creatinine measurements (n=7) are shown in Table 1.

**Table 1:** Characteristics at age 85 of the study population (n=378) and the participants who died (n=137) or refused participation before the age of 88 years (n=40) or had no four creatinine measurements between 85 and 88 years (n=7)

	**Study population (n=378)**	**population with incomplete data (n=184)**	**p**
**Socio-demographics**			
Male Sex (%)	113 (30%)	73 (40%)	<0.01
Low level of education (%)	234 (62%)	129 (70%)	<0.01
Low income (%)	194 (51%)	92 (50%)	0.62
Institutionalised	48 (13%)	51 (28%)	<0.01
**eGFR at age 85 (ml/min/1.73m²)**			
>60	169 (44%)	73 (40%)	
45-60	147 (39%)	69 (38%)	
<45	62 (16%)	42 (23%)	0.02
**Functional score (median (IQR))**			
MMSE	25 (19-25)	24 (18-24)	<0.01
GDS	2 (0-5)	2 (1-4)	0.01
ADL	12 (9-17)	13 (10-19)	<0.01

#### *eGFR and eGFR slope*

The mean eGFR at baseline (age 88) was 59 ml/min for the whole population; 45% of the participants had an eGFR > 60 ml/min, 39% had an eGFR between 45 and 59 ml/min, and 16% had an eGFR<45 ml/min. At age 88, 89 or 90 none of the participants had an eGFR<10 ml/min. The mean slope of the eGFR of the whole population was -0.4 (SD 3.1) ml/min/year. Among the subgroups identified, the mean slopes were 0.8 (SD 2.9) ml/min/year for those with an eGFR of >60 ml/min, -1.1 (SD 2.5) ml/min/year for those with an eGFR of 45–60 ml/min and -2.3 (SD 2.9) ml/min/year for those with an eGFR of <45 ml/min.

### Baseline characteristics

The baseline characteristics of the study population as a whole and for the three subgroups based on the eGFR at baseline are shown in Table 2.

**Table 2:** Baseline characteristics at age 88 of the whole study population and of the subgroups based on the eGFR (calculated by the MDRD equation)

	**all (n=378)**	**eGFR at age 88 years**			p*
		**≥60 (n=169)**	**45-59 (n=147)**	**<45 (n=62)**	
**Socio-demographics**					
Male sex (%)	113 (30%)	61 (36%)	37 (25%)	15 (24%)	0.03
Low level of education (%)	231 (61%)	95 (56%)	98 (67%)	39 (63%)	0.50
Low income (%)	194 (51%)	81 (49%)	77 (52%)	36 (58%)	0.16
Institutionalised (%)	48 (13%)	20 (12%)	13 (9%)	15 (24%)	0.07
**Comorbidity**					
Diabetes mellitus (%)	48 (13%)	25 (15%)	16 (11%)	7 (11%)	0.42
CVA in history (%)	38 (10%)	16 (10%)	15 (10%)	7 (11%)	0.68
MI in history (%)	36 (10%)	10 (6%)	14 (10%)	12 (19%)	<0.01
**Anthropometric and nutrition parameters (mean (SD))**					
Weight	68 (13)	67 (15)	69 (13)	68 (14)	0.59
Serum Albumin	41 (3)	41 (4)	41 (3)	41 (3	0.06
**Functional scores** (median (IQR))					
MMSE	25 (19-25)	25 (19-27)	26 (21-28)	24 (18-28)	0.30
GDS	2 (0–5)	3 (1-5)	1 (0–3)	2 (1-5)	0.20
ADL	12 (9-17)	13 (9-20)	11 (9-15)	14 (9-16)	0.20

*p was estimated by the Chi square test for categorical parameters, one-way ANOVA for normally distributed parameters and the Kruskal-Wallis test for non-normally distributed parameters.

The percentage of males was highest in the group with an eGFR >60 ml/min. No differences in demographic factors such as education level, income and institutionalisation or in the prevalences of diabetes mellitus and a history of CVA were observed between the three eGFR groups at baseline. A history of MI was more common in those with low eGFRs (p<0.01). The MMSE scores and GDS scores did not differ between the eGFR groups.

#### *Baseline eGFR and mortality*

From age 88 years onwards, 318 deaths; occurred in this cohort.

All participants were followed until 1/02/2010. 46 participants died at age 88–89, 41 at age 89–90, 50 at age 90–91, 37 at age 91–92, 41 at age 92–93, 36 at age 93–94, 39 at age 94–95. Of the 88 participants still alive at age 95, 28 participants died before 1/02/2010 and 60 participants aged 95–97 were still alive at this censordate. Of these deaths 108 were from cardiovascular causes, and 210 were from non-cardiovascular causes.

Compared with participants with an eGFR >60 ml/min at age 88 (reference), participants with an eGFR <45 ml/min had a similar gender adjusted total mortality risk (HR 0.96 (95% CI 0.67-1.37)) and cardiovascular mortality risk (HR 1.50 (95% CI 0.85-2.62)). In participants with an eGFR between 45 and 59 ml/min, the gender adjusted overall (HR 0.99 (95% CI 0.73-1.32) and cardiovascular (HR 1.05 (95% CI 0.62-1.78) mortality risks were similar to those of participants with an eGFR >60 ml/min.

#### *eGFR slope and mortality*

**Figure 2:**
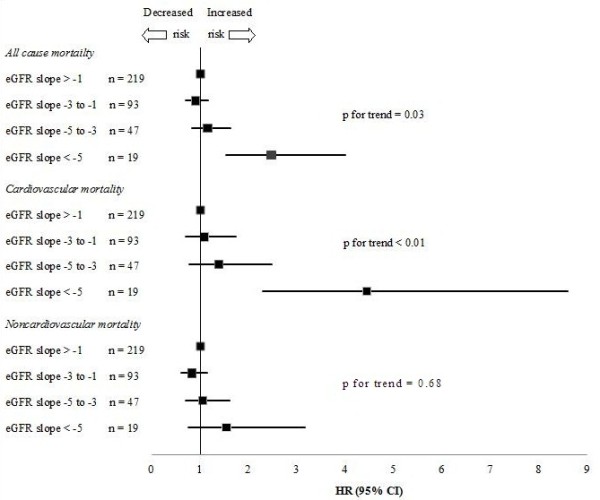
**All-cause, cardiovascular and non-cardiovascular mortality depending on the change in the eGFR per year among 378 participants aged 88 years at baseline expressed as hazard ratios (HR) adjusted for gender with 95% CIs.** Values greater than 1.0 indicate an increased risk.

Figure 2 shows the hazard ratios (adjusted for gender and the mean of the eGFRs at age 85 and 88) for total, cardiovascular and non-cardiovascular mortality for all participants according to the eGFR slope using cut-offs of -5, -3 and -1 ml/min/year. The highest total mortality and cardiovascular mortality risks were observed for participants with a decrease of 5 ml/min/year or more in the eGFR. No between-group differences in non-cardiovascular mortality were found.

#### *eGFR, eGFR slope and its relationship with mortality*

The relationship between the combination of the eGFR (in 3 subgroups) and the eGFR slope (in 4 subgroups using cut-offs of -5, -3 and -1 ml/min/year) and mortality is shown in Table 3.

**Table 3:** Relationships between both the eGFR at age 88 years and the decrease in the eGFR 3 years before (between age 85 and 88) and total and cardiovascular (CV) mortality

		**eGFR slope per year between 85 and 88 years in ml/min/1.73 m**^**2**^**/year**			
		**>-1**	**-1 to -2.99**	**-3 to -4.99**	**<-5**
**eGFR at age 88 > 60 (n=169)**		*n=128*	*n=26*	*n=12*	*n=3*
	**Overall mortality**	1 (ref)	1.37 (0.82-2.31)	2.17 (1.08-4.40)	2.63 (0.79-8.76)
	**CV mortality**	1 (ref)	1.10 (0.39-3.05)	2.99 (1.02-9.07)	8.35 (2.15-32.46)
**eGFR at age 88** **45**–**59 (n=147)**		*n=71*	*n=46*	*n=21*	*n=9*
	**Overall mortality**	1 (ref)	0.77 (0.47-1.26)	1.27 (0.69-2.35)	3.16 (1.44-6.95)
	**CV mortality**	1 (ref)	0.89 (0.38-2.07)	0.67 (0.18-2.78)	4.23 (1.22-14.65)
**eGFR at age 88 < 45 (n=62)**		*n=20*	*n=21*	*n=14*	*n=7*
	**Overall mortality**	1 (ref)	0.63 (0.31-1.26)	0.54 (0.18-1.61)	2.03 (0.59-7.02)
	**CV mortality**	1 (ref)	0.99 (0.39-2.55)	0.34 (0.04-2.76)	4.72 (1.21-18.47)

The results are presented as the HR (95% CI) and were adjusted for gender and the mean of the eGFR at ages 85 and 88. The results are shown with one reference group for each eGFR subgroup.

* No events.

Participants with eGFR >60 ml/min and eGFR decrease of ≥3 ml/min/year, participants with eGFR 45–59 ml/min and eGFR decrease of ≥5 ml/min and participants with an eGFR of <45 ml/min and an eGFR decrease of ≥5 ml/min/year had a higher total and cardiovascular mortality risk than the group of participants with an eGFR decrease of <1 ml/min/year.

Two sensitivity analyses were performed to further analyse this relation between mortality and the combination of the eGFR and eGFR decrease. A first sensitivity analysis (see Table 4) corrects for the eGFR at baseline and gender. This analysis shows similar result as Table 3.

**Table 4:** Sensitivity analysis 1 of the relationships between both the eGFR at age 88 years and the decrease in the eGFR 3 years before (between age 85 and 88) and total and cardiovascular (CV) mortality

		**eGFR slope per year between 85 and 88 years in ml/min/1.73 m²/year**			
		**>-1**	**-1 to -2.99**	**-3 to -4.99**	**<-5**
**eGFR at age 88 > 60 (n=169)**		*n=128*	*n=26*	*n=12*	*n=3*
	**Overall mortality**	1 (ref)	1.35 (0.81-2.25)	2.09 (1.07-4.09)	2.43 (0.74-8.03)
	**CV mortality**	1 (ref)	1.12 (0.41-3.08)	3.16 (1.12-8.90)	8.99 (2.34-34.60)
**eGFR at age 88 45-59 (n=147)**		*n=71*	*n=46*	*n=21*	*n=9*
	**Overall mortality**	1 (ref)	0.70 (0.43-1.15)	1.07 (0.56-2.05)	2.31 (1.06-5.06)
	**CV mortality**	1 (ref)	0.79 (0.34-1.88)	0.55 (1.39-2.12)	2.87 (0.84-9.82)
**eGFR at age 88 < 45 (n=62)**		*n=20*	*n=21*	*n=14*	*n=7*
	**Overall mortality**	1 (ref)	0.60 (0.29-1.23)	0.48 (0.16-1.40)	1.77 (0.52-6.10)
	**CV mortality**	1 (ref)	0.91 (0.34-2.39)	0.31 (0.04-2.42)	4.00 (1.04-15.43)

The results are presented as the HR (95% CI) and were adjusted for the eGFR at age 88 and gender. The results are shown with one reference group for each eGFR subgroup.

* No events.

Second the relation between the participant’s weight and changes in weight and eGFR changes were analyzed since (changes in) weight correlates to (changes in) muscle mass which correlates with the serum creatinine level. No relation was found between decrease in eGFR between 85 and 88 years and weight at age 88 (p for trend 0.56) or change in weight (p for trend 0.15) between 85 and 88 years. In a second sensitivity analysis (see Table 5) change in weight between age 85 and 88, a MI in the history and gender were used as co- variables. This second sensitivity analysis showed similar results as Table 3 with even higher HR for the high risk groups

**Table 5:** Sensitivity analysis 2 for the relationships between both the eGFR at age 88 years and the decrease in the eGFR 3 years before (between age 85 and 88) and total and cardiovascular (CV) mortality

		**eGFR slope per year between 85 and 88 years in ml/min/1.73 m²/year**			
		**>-1**	**-1 to -2.99**	**-3 to -4.99**	**<-5**
**eGFR at age 88 > 60 (n=169)**		*n=128*	*n=26*	*n=12*	*n=3*
	**Overall mortality**	1 (ref)	1.44 (0.85-2.44)	2.77 (1.35-5.68)	4.41 (1.27-15.33)
	**CV mortality**	1 (ref)	1.32 (0.48-3.67)	4.08 (1.39-11.96)	10.35 (2.46-43.61)
**eGFR at age 88 45-59 (n=147)**		*n=71*	*n=46*	*n=21*	*n=9*
	**Overall mortality**	1 (ref)	0.89 (0.53-1.52)	1.58 (0.84-2.96)	3.37 (1.51-7.52)
	**CV mortality**	1 (ref)	0.95 (0.38-2.37)	0.83 (0.22-3.09)	4.41 (1.24-15.78)
**eGFR at age 88 < 45 (n=62)**		*n=20*	*n=21*	*n=14*	*n=7*
	**Overall mortality**	1 (ref)	0.74 (0.36-1.51)	0.50 (0.17-1.46)	2.26 (0.64-8.03)
	**CV mortality**	1 (ref)	0.97 (0.37-2.54)	0.29 (0.04-2.32)	4.04 (1.01-16.20)

The results are presented as the HR (95% CI) and were adjusted for the change in weight between 85 and 88 years, history of MI and gender. The results are shown with one reference group for each eGFR subgroup.

* No events.

#### *eGFR, eGFR slope and functional performance*

The mean changes in the functional scores (MMSE, GDS and ADL) between 88 and 90 years for the study population divided into subgroups based on the eGFR (3 subgroups), on the eGFR slope (4 subgroups using cut-offs of -5, -3 and -1 ml/min/year) and on a combination of the two are shown in Table 6. No relationship was observed between the baseline eGFR, the eGFR slope or a combination of the two and the mean change in the MMSE, GDS or ADL score between the age of 88 and 90.

**Table 6:** Relationships between the baseline eGFR (at age 88 years), the eGFR slope (between age 85 and 88 years) and a combination of these two parameters and changes in the MMSE, GDS and ADL scores between age 88 and 90 years

**All participants**		**eGFR slope per year between 85 and 88 years in ml/min/1.73 m²/year**				**P for the eGFR slope per year**
		**>-1**	**-1 to -2.99**	**-3 to -4.99**	**<-5**	
Mean change in the MMSE score (+SE)						
All participants (n=274)	-1.59 (0.22)	-1.30 (0.28)	-1.74 (0.40)	-2.39 (0.63)	-2.55 (1.49)	*0.33*
eGFR 88 > 60	-1.30 (0.30)	-1.09 (0.35)	-2.31 (0.75)	-2.00 (1.41)	-1.50 (1.5)	*0.27*
eGFR 88: 45-60	-1.55 (0.32)	-1.42 (0.45)	-1.14 (0.54)	-2.38 (0.99)	-3.14 (1.42)	*0.39*
eGFR 88 < 45	-2.66 (0.77)	-2.33 (1.71)	-2.62 (0.99)	-2.75 (1.22)	-4.50 (7.50)	*0.95*
*P (trend) for eGFR subgroups*	*0.13*	*0.50*	*0.27*	*0.92*	*0.46*	
Mean change in the GDS score (+SE)						
All participants (n=198)	0.68 (0.20)	0.82 (0.30)	0.51 (0.24)	0.70 (0.68)	-0.40 (1.12)	*0.76*
eGFR 88 > 60	1.05 (0.36)	1.09 (0.45)	1.14 (0.64)	1.00 (1.41)	-1.00 (3.00)	*0.86*
eGFR 88: 45-60	0.50 (0.24)	0.59 (0.43)	0.37 (0.30)	0.67 (0.56)	0.00 (1.00)	*0.95*
eGFR 88 < 45	-0.5 (.59)	-0.50 (0.56)	0.10 (0.43)	0.33 (3.93)	/	*0.88*
*P (trend) for eGFR subgroups*	*0.22*	*0.43*	*0.33*	*0.96*	*0.72*	
Mean change in the ADL score (+SE)						
All participants (n=274)	3.58 (0.35)	3.88 (0.48)	3.12 (0.64)	3.42 (1.04)	3.0 (1.17)	*0.79*
eGFR 88 > 60	3.77 (0.56)	4.04 (0.67)	2.95 (1.35)	2.86 (1.67)	2.50 (2.50)	*0.86*
eGFR 88: 45-60	3.05 (0.59)	3.24 (0.68)	2.57 (0.72)	2.44 (1.62)	3.43 (1.77)	*0.89*
eGFR 88 < 45	5.05 (1.10)	5.42 (2.18)	4.75 (1.87)	5.88 (1.89)	2.00 (1.00)	*0.91*
*P (trend) for eGFR subgroups*	*0.12*	*0.49*	*0.42*	*0.39*	*0.90*	

Of the 378 participants at age 88 years, 87 died before age 90 and 17 refused to participate further. The GDS scores were not available for 76 participants who had at least once a low MMSE score in the annual measurements between 88 and 90 years old.

## Discussion

The present study shows that combining the eGFR and the eGFR slope allows the identification of very elderly with a highly increased mortality risk in the general population. The highest mortality in every eGFR group was found in the participants with the largest decrease in the eGFR. Adjustment for changes in weight over time, as proxy for changes in muscle mass with correlates well with serum creatinine values, did not change the results.

Our findings confirm the results of earlier studies [10,19] in younger populations that showed that the subgroup of participants with the largest decrease in the eGFR had the highest mortality rates.

Interestingly, analysing our results in subgroups provided more detailed insight into the relationships between the eGFR and the slope of the eGFR on mortality in the very elderly. Participants in our study with an eGFR of ≥60 ml/min and a decrease in eGFR of >3 ml/min/year had a more than twofold higher cardiovascular mortality risk than participants with normal eGFR and a decrease of < 1 ml/min/year. Therefore, eGFR decrease could be used in older persons without CKD to identify individuals with a higher risk of cardiovascular mortality.

Among participants with eGFRs of 45–60 ml/min, only those with an eGFR decrease of 5 ml/min/year (6% of the persons with eGFRs of 45–60 ml/min) or more over 3 years had an increased mortality risk. Possible hypotheses for these differences in thresholds could be that a decrease of 3 ml/min /year or more in persons with eGFR>60 ml/min is reflecting on-going cardiovascular damage whereas a decrease of 1–5 ml/min/year in persons with eGFR<60ml/min could be explained as fluctuation in eGFR due to adaptation instead of on-going cardiovascular damage.

These results show that only a very small subgroup of the participants with an eGFR of 45–60 ml/min had an increased mortality risk. The current CKD classification [15] classifies all patients with an eGFR of 45–60 ml/min as having moderate CKD. Based on the results of our study, one may wonder whether very elderly individuals with an eGFR of 45–60 ml/min really should be labelled as having “moderate stage 3 chronic kidney disease”, as indicated in the currently used classification of CKD [15].

Earlier studies [7,8] showed that all patients with an eGFR of <45 ml/min had an increased (cardiovascular) mortality risk. A recent meta-analysis by Hallan et al. [20] showed that lower eGFR is independently associated with mortality and ESRD in all age groups. However, in older age mortality showed lower relative risk, but higher absolute risk differences. In our study, 94% (138/147) of the participants with eGFRs of 45–60 ml/min and 89% (55/62) of the participants with eGFRs of <45 ml/min had no increase in total or cardiovascular mortality compared with the participants with eGFR>60 ml/min and eGFR slope >-1 ml/min/year. This finding is relevant since it seems possible to select high risk and low risk older persons based on their eGFR and eGFR decrease, both easy to measure. Further research should investigate the benefit of interventions (like cardiovascular risk management or referral to a nephrologist) in the very elderly and higher mortality risk.

Finally, earlier research showed that there is an association between a low eGFR and more rapid cognitive decline [21], frailty [22] and ADL disability [11,12]. In this study, no relationship was found between the eGFR, the decrease in the eGFR or a combination of the two and a change in the MMSE, GDS or ADL score. It is unclear why the baseline eGFR combined with the eGFR slope predicted mortality but not changes in functional performance. One possible reason for this could be the small number of participants in some subgroups in our study. However, earlier analyses in the Leiden 85-plus Study cohort [23,24] showed significant differences in functional performance over time using the same functional performance scores that were used in this study. Therefore, we consider it unlikely that the lack of association could be explained by the use of measurements scales that were not sensitive to small changes between the age of 88 and 90 Another possible explanation is that participants with a rapid decrease in the eGFR die before developing functional decline.

### Strengths and weaknesses

One strong point of this study is that it was a prospective study with a cohort of very old persons in a population-based setting with long, almost complete, follow-up. Another strength is that the eGFR slopes were calculated using four eGFR values from every patient. Thus, the well documented effect [25] of biological and analytical variance on the slope of the eGFR was minimised. Another strong point is that we did not use only one cut-off of -3 ml/min/year but rather analysed the relationship between the eGFR slope and mortality using 2 additional cut-offs (-5 and -1 ml/min year), allowing us to differentiate a limited decrease from larger decrease in the eGFR.

One limitation of the study is that the eGFR was estimated with the 4-variable MDRD equation. This equation is currently recommended in most international guidelines and a reflection of general practice, [26] but is inadequately validated in patients age 80 and older[27]. The CKD_EPI creatinine based equation [28] which needs Isotope Dilution Mass Spectrometry (IDMS) is slightly better in estimating the eGFR in older persons [29,30]. However, in this study we did not perform the creatinine measurements with the required IDMS [31] but the Jaffe method, which was recommended at the time the data collection started. Since the MDRD equation uses non-IDMS creatinine to estimate the eGFR, the MDRD equation was used in this study. However, because the same creatinine assay was used for all repeated measurements for each individual participant in the study, the effect of using this method to determine the eGFR slope within individuals is likely limited.

In addition, a limitation of our study is its relatively small sample size, especially of some subgroups.

## Conclusions

In this study, we found that a rapid decrease in the eGFR is a predictor of mortality in older persons in the general population The combination of the baseline eGFR and the eGFR slope allowed the identification of subgroups of very elderly with higher mortality risks and subgroups of very elderly with an eGFR of <60 ml/min/year that do not have an increased risk of mortality. The combination of the eGFR and the eGFR slope did not predict a decline in functional performance.

## Competing interests

The authors declare that they have no competing interests.

## Authors’ contributions

JG had full access to all of the data and is responsible for the integrity of the data and the accuracy of the analysis. JG contributed to the study concept and design and obtained funding for the study. JG supervised the study. WDE and JG contributed to the data acquisition. All of the authors contributed to the analysis and interpretation of the data. GVP, WDE and JD conducted the statistical analyses. GVP drafted the manuscript, and all of the authors made critical revisions for important intellectual content and approved the manuscript.

## Pre-publication history

The pre-publication history for this paper can be accessed here:

http://www.biomedcentral.com/1471-2318/13/61/prepub
